# *Brucella* spp. Lumazine Synthase Induces a TLR4-Mediated Protective Response against B16 Melanoma in Mice

**DOI:** 10.1371/journal.pone.0126827

**Published:** 2015-05-14

**Authors:** Andrés H. Rossi, Ana Farias, Javier E. Fernández, Hernán R. Bonomi, Fernando A. Goldbaum, Paula M. Berguer

**Affiliations:** Fundación Instituto Leloir, IIBBA, Consejo Nacional de Investigaciones Científicas y Técnicas (CONICET), Buenos Aires, Argentina; University of Alabama at Birmingham, UNITED STATES

## Abstract

*Brucella* Lumazine Synthase (BLS) is a highly immunogenic decameric protein which can accept the fusion of foreign proteins at its ten N-termini. These chimeras are very efficient to elicit systemic and oral immunity without adjuvants. BLS signaling via Toll-Like Receptor 4 (TLR4) regulates innate and adaptive immune responses, inducing dendritic cell maturation and CD8+ T-cell cytotoxicity. In this work we study the effect induced by BLS in TLR4-expressing B16 melanoma. In order to evaluate the effectiveness of BLS as a preventive vaccine, C57BL/6J mice were immunized with BLS or BLS-OVA, and 35 days later were subcutaneously inoculated with B16-OVA melanoma. BLS or BLS-OVA induced a significant inhibition of tumor growth, and 50% of mice immunized with the highest dose of BLS did not develop visible tumors. This effect was not observed in TLR4-deficient mice. For treatment experiments, mice were injected with BLS or BLS-OVA 2 days after the inoculation of B16 cells. Both treatments induced significant and equal tumor growth delay and increased survival. Moreover, BLS and BLS-OVA stimulation were also effective in TLR4-deficient mice. In order to study whether BLS has a direct effect on tumor cells, B16 cells were preincubated with BLS, and after 48h, cells were inoculated. Tumors induced by BLS-stimulated cells had inhibited growth and survival was increased. In the BLS group, 40% of mice did not develop tumors. This effect was abolished by the addition of TLR4/MD2 blocking antibody to cells before BLS stimulation. Our work demonstrates that BLS immunization induces a preventive antitumor response that depends on mice TLR4. We also show that BLS generates a therapeutic effect in mice inoculated with B16 cells. Our results show that BLS acts directly in cultured tumor cells via TLR4, highly suggesting that BLS elicits its therapeutic effects acting on the TLR4 from B16 melanoma cells.

## Introduction

Vaccines for antitumor therapies or for the prevention of neoplasia are now in a stage of incipient development. There are many biomolecules capable of potentiate the immune response when co-administered with the antigen of interest, but only a few adjuvants have been approved for its use in medicine due to their toxicity. Toll-Like Receptor (TLR) agonists are of particular interest in this area because they induce the activation of dendritic cells (DC), promote Th1-type immune responses, antigen presentation and cytotoxicity, all of which are important factors in the development of antitumor immunity [1–5]. TLR4 is particularly important for development of a strong adaptive immune response by stimulation of the antibody class switching, affinity maturation, and formation of memory cells [6]. Additionally, it has been reported that TLR4 expression by DC is a prerequisite for efficient antigen presentation of tumor antigens provided by dying cancer cells [7].

The role of TLRs in tumor development and in cancer vaccine responses is still not fully understood. Clinical and preclinical studies show that existing vaccination protocols can be improved by the co-administration of TLR agonists [8–10]. The usage of high doses of these agonists usually has toxic effects, and in some cases, TLR stimulation can also result in enhanced regulatory T cell proliferation and suppressor function by inhibiting NK cell cytotoxicity, favoring tumor development [11–14].

In recent years it has been reported that TLR expression is not only limited to immune cells but rather TLRs are expressed by tumor cells from different origins, both in human and mice. Tumors exhibiting elevated TLR expression include breast, colorectal, melanoma, lung, prostate, glioma, pancreatic, liver, and esophageal cancers [15–19]. Studies have correlated elevated TLR expression and dysfunctional immunity within the tumor microenvironment with cancer progression and reduced patient survival in a number of solid tumors [16, 20–22]. In human melanoma it has been reported that high TLR4 expression is associated with a shortened relapse-free survival [23]. Also, human myeloma cells express a broad range of TLRs, and triggering TLR7 and TLR9 induces tumor cell growth and prevents chemotherapy-induced apoptosis [24]. These studies are of relevance because the level of TLR expression in tumors could be used to predict the outcome of the disease and the success of potential treatments.

Bacillus Calmette-Guerin (BCG) has been used successfully for the treatment of bladder cancer for more than 3 decades. Monthly BCG maintenance therapy improves recurrence-free 5-year cumulative survival rate [25]. BCG promotes dendritic cell maturation, and this effect is TLR4 as well as TLR2 dependent [26]. Furthermore, BCG can induce expression of TNF related apoptosis-inducing ligand (TRAIL) on tumor infiltrating dendritic cells, therefore rendering them cytotoxic against tumor cells [27]. Another successful case in the use of TLR agonists in cancer treatment is the TLR7 ligand imiquimod, approved for the topical treatment of skin basal cell carcinoma with curative effects in a majority of patients which has been linked to activation of innate and adaptive antitumor immune mechanisms [28–30]. The combined use of TLR agonists with therapeutic cancer vaccines or other chemotherapeutics that prime the immune system for the development of Th1 cytotoxic responses against tumor antigen-expressing cells has yielded promising results. It has also been shown that TLR ligands can enhance antitumor immunity in radio and chemotherapy [7].

The enzyme Lumazine Synthase from *Brucella abortus* (BLS) is a highly stable decameric protein [31, 32]. It is possible to insert foreign peptides or proteins at its ten-amino acid termini [33] and these chimeras are very efficient in generating oral and systemic immunity, even in the absence of adjuvants, which are commonly needed in the formulation of subunit-based vaccines. BLS has been extensively used as a carrier for peptides and proteins being a proven successful platform for antigen presentation to the immune system for vaccine development [34–36]. We have previously shown that BLS activates dendritic cells via TLR4, inducing the upregulation of costimulatory molecules and the secretion of proinflamatory cytokines and several chemokines [37]. BLS induces the cross presentation of covalently attached peptides and generates a strong and long-lasting humoral immune response without adjuvants [38].

B16 melanoma is a syngeneic murine melanoma derived from a spontaneously arising melanoma of C57BL/6J origin [39, 40] and it is the most frequently used melanoma model in hosts with intact immune system. In this work we use a B16-F1 cell line that expresses OVA in a non-secreted form (B16-OVA, [41]). Human melanoma is highly curable if detected in its earliest stages and treated properly; but the survival time for patients with metastatic melanoma averages 3–15 months [42, 43]. Hence, early diagnosis and surgical removal of the primary tumor provide the best opportunities for cure or prolonged survival to patients [44–46]. Currently, there are only a few available treatments, including surgery, chemotherapy [47, 48], IL-2 and/or IFN-α immunotherapy [49] and radiotherapy [50]. Unfortunately, there is no satisfactory treatment for metastatic melanoma due to its resistance to current chemotherapy and immunotherapy regimens [51]. Other treatments that impact on different immunomodulatory mechanisms, to induce an immune response against the tumor, have emerged in the last years: anti-PD-1 and anti-CTLA4 [52, 53], BRAF and MEK inhibitors [54–56], and others. The inhibition of melanogenesis has also been proposed as an adjuvant strategy in the treatment of melanotic melanomas [57–60], since melanin enhances an immunosupressive environment and protects the cells from radiotherapy [58], attenuating the treatment effect. Some DC-based vaccines strategies have employed DC to enhance specific immunity in preclinical models and in clinical studies [61–65]. Although results from many clinical studies have been very encouraging, treatment of metastatic melanoma remains challenging due to the difficulty to obtain long-lasting clinical benefits, even with the novel approved drugs. There is increasing evidence that combination therapies would generate a more effective response in a broader spectrum of patients.

In this work we evaluate the effect of BLS stimulation in B16 melanoma growth in mice and also whether it has a direct effect on B16 cells. The results show that BLS elicits a protective role in mice against B16 melanoma, slowing tumor growth and prolonging mice survival. These effects are observed when mice are immunized before tumor cells are injected and also as a treatment, when mice are inoculated with BLS 2 days after tumor injection. The preventive effect is dependent on mice TLR4 and the therapeutic effect is probably dependent only on tumor TLR4. We demonstrate that stimulating B16 cells with BLS *in vitro*-before its inoculation- significantly augments survival and that this effect is abolished when tumor cells are pretreated with TLR4/MD2 monoclonal antibody. BLS signaling via TLR4 could contribute to the success of cancer treatment in combination therapies.

## Materials and Methods

### Protein purification

Cloning, recombinant expression, and purification of BLS protein were performed as described previously [33, 66]. Briefly, the BLS gene was cloned into the pET11a vector (Novagen, Madison, USA) and transformed and expressed as inclusion bodies in the BL21 (DE3) strain of *Escherichia coli*. The inclusion bodies were solubilized in 50 mM Tris, 5 mM EDTA, and 8 M urea (pH 8.0) overnight at room temperature with agitation. The solubilized material was refolded by dialysis against PBS containing 1 mM DTT for 72 h. This preparation was purified with a Q-Sepharose column (Amersham Biosciences, Little Chalfont, UK) in a fast performance liquid chromatography apparatus (Amersham Biosciences) using a linear gradient of NaCl between 0 and 1 M in 50 mM Tris (pH 8.5). The peak enriched with BLS was further purified on a Superdex-200 column with PBS, 1 mM DTT. The purity of the BLS preparation was determined using 15% (w/v) SDS-PAGE. BLS was concentrated (to 7 mg/ml), frozen in liquid N_2_, and stored at −20°C. Purified BLS was detoxified by incubation with 1 mg of BLS with 500 μl of polymyxin B-agarose (PMB-agarose, Sigma-Aldrich, Saint Louis, MO, USA) overnight twice at 4°C, as previously described [37]. The procedure performed to generate BLS chimeras was previously described [33]. To generate BLS-OVA, the coding sequence for chicken OVA peptide 257–264 was inserted at the N terminus of BLS in vector pet11a. The resulting vector was transformed into and expressed in BL21 (DE3) *E*. *coli*. The chimera was purified from bacterial cytoplasm. The purification steps were the same as those for BLS. The purity of the samples was determined by SDS-PAGE. BLS-OVA was detoxified with PMB-agarose as described for BLS. Limulus amebocyte lysate (LAL) test was performed in order to assure that BLS and BLS-OVA preparations were free of LPS. Determinations were carried out following manufacturer’s instructions (Associates of Cape Cod. Rev 002. Nov 2003. LAL Pyrotell Multitest vial instruction sheet). Pyrotell LAL for gel-clot assay, LAL reagent water, endotoxin standard, tips and tubes were purchased from Associates of Cape Cod (Woods Hole, MA, USA).

### Mice and Cell Culture

C57BL/6J mice and C57BL/10ScNJ mice (carrying a spontaneous deletion of the Tlr4 gene) were obtained from The Jackson Laboratory and bred in the animal facility at Leloir Institute. All mice were bred under specific pathogen-free conditions and were used at 8–10 week of age. B16-F1 melanoma (ATCC CRL-6323), syngeneic from C57BL/6 mice was a kind gift from Dr José Mordoh´s lab and was cultured at 37°C under 5% CO_2_ in endotoxin-free RPMI 1640 medium supplemented with 10% FBS (Gibco; Grand Island, NY, USA), penicillin and streptomycin, 1 mM pyruvate and 4 mM L-glutamine. OVA-expressing B16-F1 melanoma (B16-OVA) was kindly provided by Dr Paolo Dellabona [41] and was cultured in the same media with the addition of 100 μg/ml hygromycin B (Roche Diagnostics, Mannheim, Germany).

### Tumor Volume

Tumor growth was monitored every 2 or 3 days and diameters were measured using a caliper. The major longitudinal diameter (length) and the major transverse diameter (width) were determined and tumor volume was approximated based on caliper measurements by the following formula: Tumor volume = 0.5 × (length × width^2^).

### Vaccination and Melanoma Inoculation

In the preventive vaccination assays, C57BL/6J and C57BL/10ScNJ mice were immunized with 100 or 200 μg of BLS or 100 μg of BLS-OVA in PBS subcutaneously in the base of the tail. After 35 days, mice were inoculated with 2.5x10^5^ B16 or B16-OVA melanoma cells subcutaneously in the right flank. In the treatment assays, mice were inoculated with 2.5x10^5^ B16 or B16-OVA cells and 2 or 10 days later were sc injected with 100 or 200 μg of BLS or 100 μg of BLS-OVA. To study BLS direct effects on tumor cells, B16 cells were preincubated *in vitro* with 100 μg of BLS or 5 ng of LPS. After 48h cells were washed 3 times with RPMI and 2.5x10^5^ cells were sc inoculated in the right flank of C57BL/6J and C57BL/10ScNJ mice. To block TLR4, cells were incubated with 1 μg of TLR4/MD2 monoclonal antibody (raised in rat, clone MTS510; BD Pharmingen, San Diego, CA, USA) for 8h. Cells were then washed 3 times with RPMI and incubated with BLS as described.

### Apoptosis Assay

Induction of apoptosis was evaluated following Annexin V staining of adherent cells protocol for flow cytometry. Briefly, B16 cells were cultured in a 6-well plate (2.5x10^5^cells/well) in 2 mL standard cell culture medium. After 18h medium was replaced and 100 μg of BLS were added. Forty eight hours later, apoptosis was assessed using the Annexin V-PE/7-AAD detection kit (#559763, BD Pharmingen, San Diego, CA, USA). Cells were gently detached using Accutase Cell Detachment Solution (BD Biosciences; San José, CA, USA) and fluorescence-activated cell sorter analysis was performed, as described below.

### Flow Cytometry

Cells were stained with the following mAbs (BD Pharmingen, San Diego, CA, USA) and subjected to FACS analysis: FITC-conjugated anti-CD80 (Hamster, clone 16-10A1), and PE-conjugated anti-TLR4/MD2 (Rat, clone MTS510). Cells were acquired on a FACScan cytometer (BD Biosciences, San José, CA, USA) and data were analyzed by using CellQuest software (BD Immunocytometry Systems, San José, CA, USA).

### Ethics Statement

This study was carried out in strict accordance with the recommendations from the Guide for the Care and Use of Laboratory Animals of the National Institutes of Health. The protocol was approved by the Committee on the Ethics of Animal Experiments of the Leloir Institute (Protocol #FG58/2011). All efforts were made to minimize suffering. Mice were monitored at least every 2 or 3 days and sacrificed by cervical dislocation when tumors reached a volume greater than 3000 mm^3^, when tumors where ulcerated or signs of discomfort were observed. A small number of animals (2–3 per experiment) inoculated with B16 cells were found dead without previous signs of poor clinical condition. A minimum of 6 and a maximum of 10 mice were used per group for each experiment; the total number of mice used for this work was approximately 280.

### Statistical Analysis

GraphPad PRISM 5.0 software (GraphPad) was used for statistical analyses. All the experiments were carried out in duplicate or triplicate and data was pooled. Statistical significance was set at p<0.05 (* = p< 0.05, ** = p< 0.01 and *** = p< 0.001). Survival curves were estimated using a Kaplan-Meier plot and compared using the log-rank test. FACS results are expressed as means ± SD; tumor volumes are shown as means ± SEM. Levels of significance were determined using unpaired two-tailed Student's t-test.

## Results

### Protective effect of BLS against B16 melanoma

**Fig 1:**
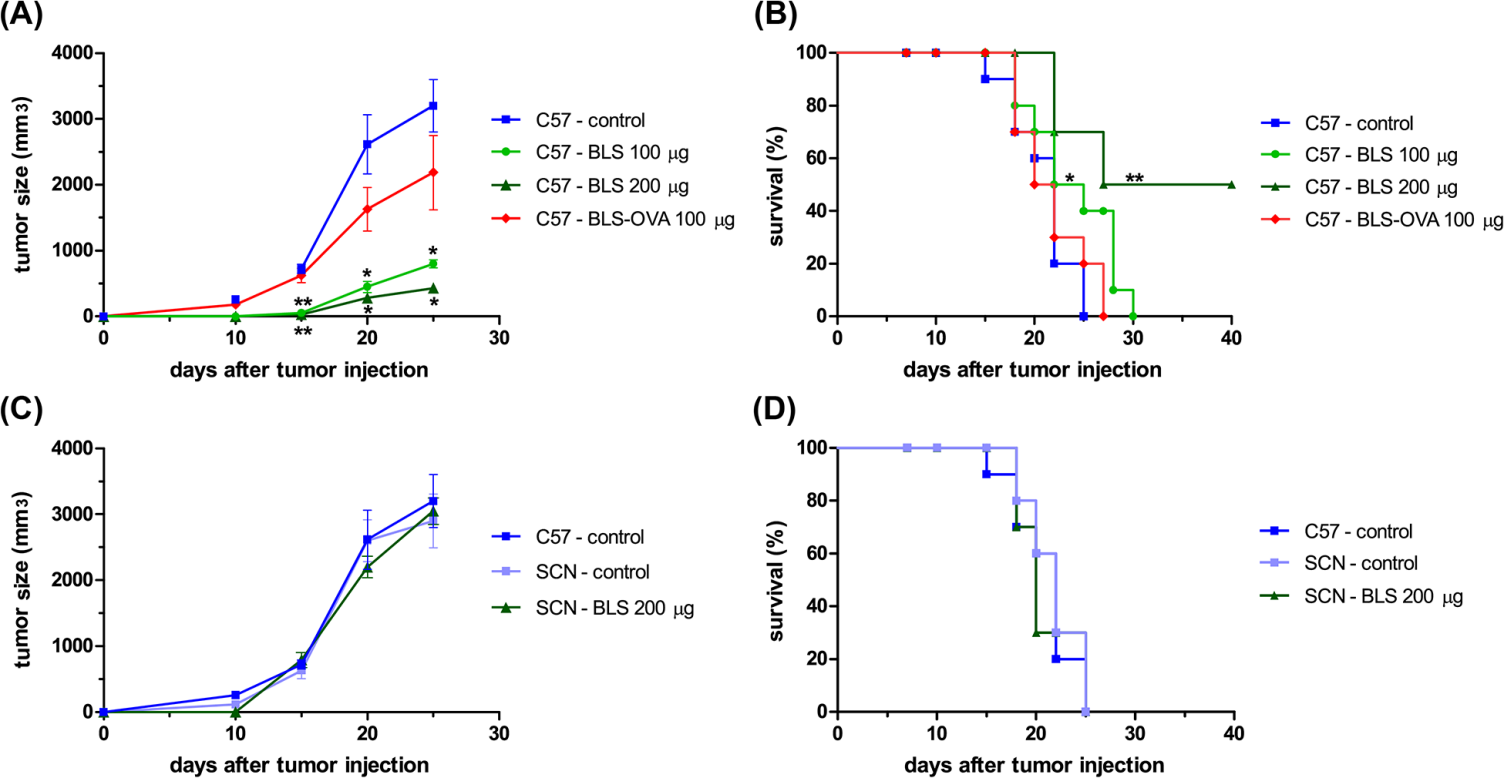
BLS immunization induces a protective effect against B16 melanoma. C57Bl/6J (C57, (A) and (B)) and C57BL/10ScNJ mice (SCN, (C) and (D)) were immunized with 100 or 200 μg of BLS or 100 μg of BLS-OVA in PBS or left untreated (control) subcutaneously in the base of the tail. After 35 days, all mice were inoculated with 2.5x10^5^ B16 melanoma or B16-OVA cells subcutaneously in the right flank. Tumor growth was monitored and diameters were measured using a caliper; Tumor volume was estimated as ½ (length × width^2^). (A) and (C) show tumor growth; (B) and (D) show the survival rate. Data from two independent experiments with B16-OVA cells have been pooled (5 mice per group). n = 10, * p<0.05, ** p<0.01.

We have previously shown that one dose of BLS without adjuvants, induces a strong innate immune response via TLR4 in mice. To test whether this response is capable of preventing or slowing tumor growth, C57BL/6J mice were immunized with a single dose of 100 or 200 μg of BLS and 35 days later were sc injected with B16 melanoma. Non-immunized control mice developed visible tumors at 8–10 days; in contrast, immunized mice showed a significant inhibition of tumor growth where 50% of the mice immunized with a 200 μg dose, did not develop visible tumors over a 90-day follow up. Fig 1A shows tumor growth and Fig 1B shows the survival rate. To evaluate the dependence of this protective effect on TLR4, the same experiments were conducted in TLR4-deficient C57BL/10ScNJ mice. Immunization with BLS did not induce effects in tumor growth in TLR4 deficient mice, in contrast to the same treatment in wild type mice (Fig 1C and 1D). These results show that BLS elicits an immunoprotective response against B16 melanoma growth via TLR4. As we have shown in a previous work, BLS induces the cross presentation of covalently attached peptide OVA_257-264_ and this chimera induces rapid activation of CTLs and a specific cytotoxic response in a TLR4-dependent manner. We therefore evaluated if specific response towards tumor antigens could contribute to the protective effect of BLS. To this end, mice were first immunized with the chimera BLS-OVA-consisting in BLS protein decorated with 10 copies of the peptide OVA_257-264_- and 35 days later were injected with an OVA-expressing B16 melanoma (B16-OVA). Surprisingly, BLS-OVA immunization induced a protective effect in tumor growth but to a lesser extent than BLS (Fig 1). This result suggests that the specific response does not generate a contribution to the overall protective response. The reason of the obtained differences between BLS and BLS-OVA in protection remains to be studied. In our hands, the level of BMDC activation induced by BLS and BLS-OVA do not show significant differences, inducing similar levels of costimulatory molecules (S1 Fig).

### Therapeutic effect of BLS vaccination

**Fig 2:**
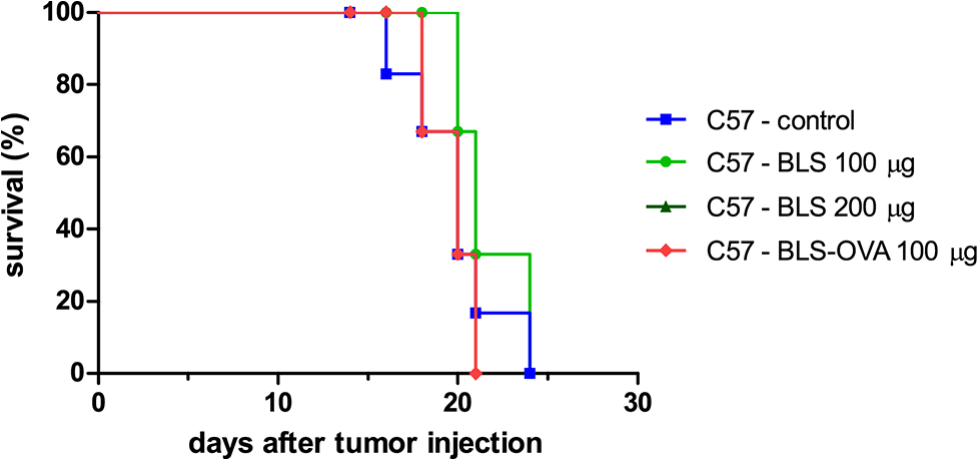
BLS stimulation does not protect mice with B16 melanoma at 10 days of tumor inoculation. C57Bl/6J mice were inoculated with 2.5x10^5^ B16-OVA cells sc in the right flank; 10 days later, mice were injected sc in the base of the tail with 100 μg of BLS or BLS-OVA or left untreated (control). Tumor growth was monitored; curves show survival rate; n = 6.

**Fig 3:**
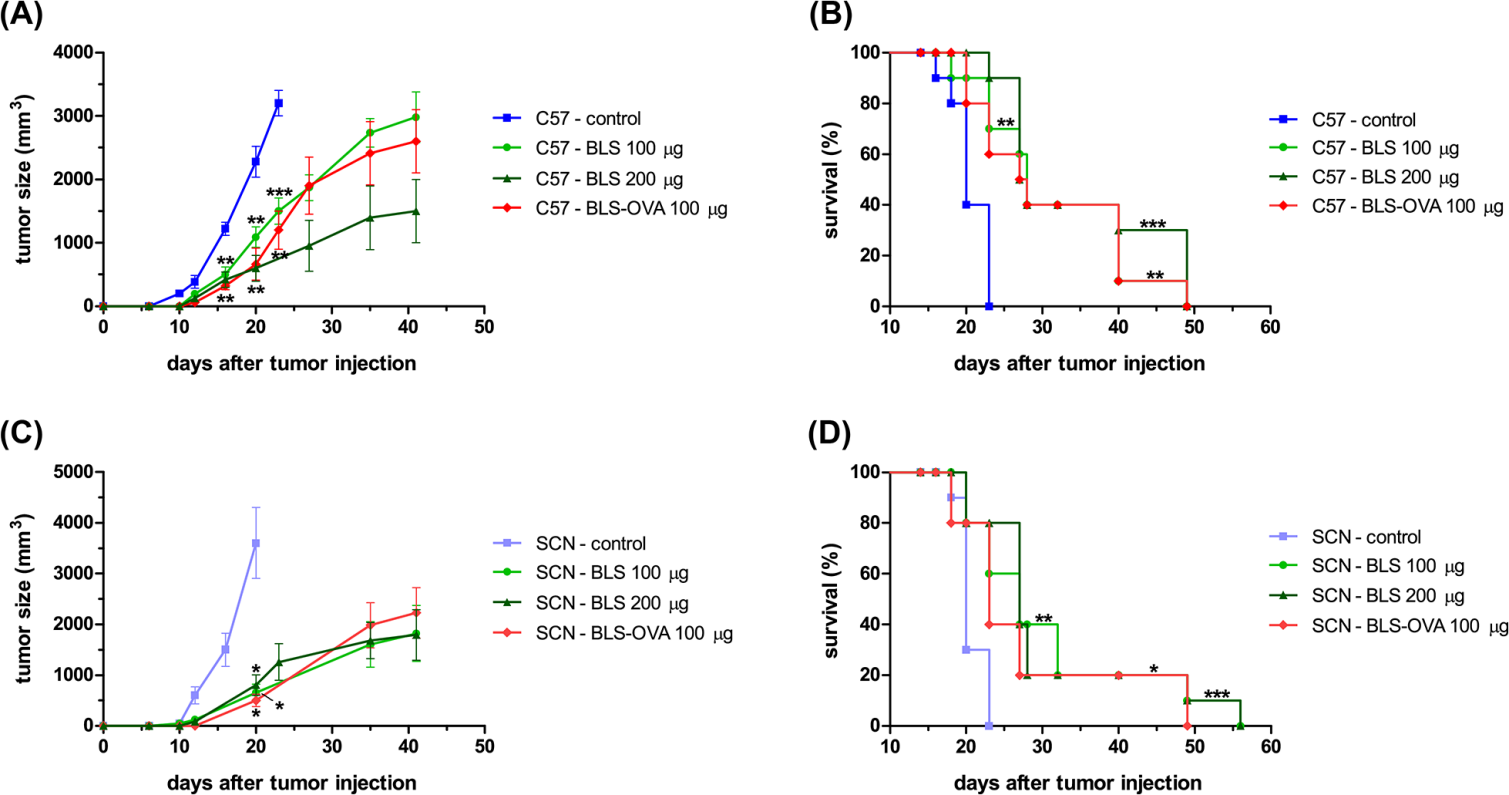
BLS induces a therapeutic effect in mice with B16 melanoma at 2 days of tumor inoculation. C57Bl/6J (C57, (A) and (B)) and C57BL/10ScNJ mice (SCN, (C) and (D)) were inoculated with 2.5x10^5^ B16 melanoma or B16-OVA cells and 2 days later were immunized with 100 or 200 μg of BLS or 100 μg of BLS-OVA or left untreated (control). Tumor growth was monitored and diameters were measured using a caliper; Tumor volume = ½ (length × width^2^). (A) and (C) show tumor growth; (B) and (D) show the survival rate. Data from two independent experiments with B16-OVA cells have been pooled (5 mice per group). n = 10, * p<0.05, ** p<0.01 and *** p< 0.001.

**Fig 4:**
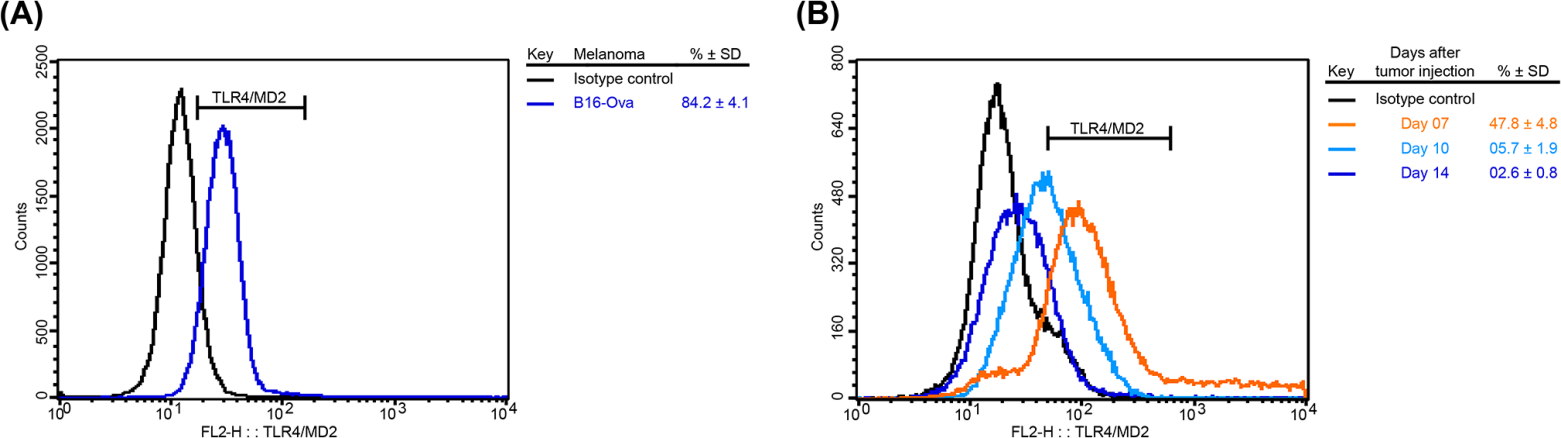
TLR4 expression decreases in B16-OVA tumors. TLR4/MD2 expression was determined in (A): cultured B16-OVA cells and (B): excised tumors from C57Bl/6J mice at different times post-B16-OVA sc inoculation. Representative histograms from 3 independent experiments are shown (n = 4).

Once the partially protective effect of BLS against B16 melanoma was demonstrated, we decided to evaluate its therapeutic potential. For these experiments, mice were sc injected with BLS 2 or 10 days after the inoculation of B16 cells. Stimulation with BLS at day 10 induced a delay in tumor growth, however, it was not significant and survival was not increased (Fig 2). BLS treatment at day 2 produced a significant delay in tumor growth with both doses assayed (Fig 3A). In accordance with this result, it was observed a significant increase in mice survival (Fig 3B). These results show that BLS has a therapeutic protective role in mice with B16 melanoma, generating a protective response at the first stages of tumor growth but not at later stages. In contrast with what we observed in the preventive assays, in TLR4 deficient mice BLS induced a similar protection than in wild type mice. This result demonstrates that the protective therapeutic effect is not dependent on mice TLR4 and suggests that BLS may act directly on the TLR4 on B16 cells. Fig 3C shows tumor growth and Fig 3D shows the survival rate in TLR4 deficient mice. No statistical differences were observed between immunization with BLS-OVA or with BLS in mice sc injected with B16-OVA cells in both strains. The effect induced by BLS is not augmented by coupling a tumor antigen to its structure in the experimental conditions assayed. In order to explain the lack of therapeutic effect when BLS is administered 10 days after the inoculation of the tumor, the evolution of TLR4 expression in B16-OVA cells was studied: the expression of tumor TLR4/MD2 was analyzed by FACS at different days after B16 melanoma inoculation in mice (Fig 4). TLR4/MD2 was expressed in 84% of B16-OVA cells before the inoculation; in 7-day tumors it was expressed in 47.8% of the cells and this percentage dropped to 5.7% at day 10; 4.2% at day 12 and 2.6% at day 14. These results clearly show that the expression of surface TLR4/MD2 diminishes over time *in vivo*.

### BLS impacts directly on B16 cells

**Fig 5:**
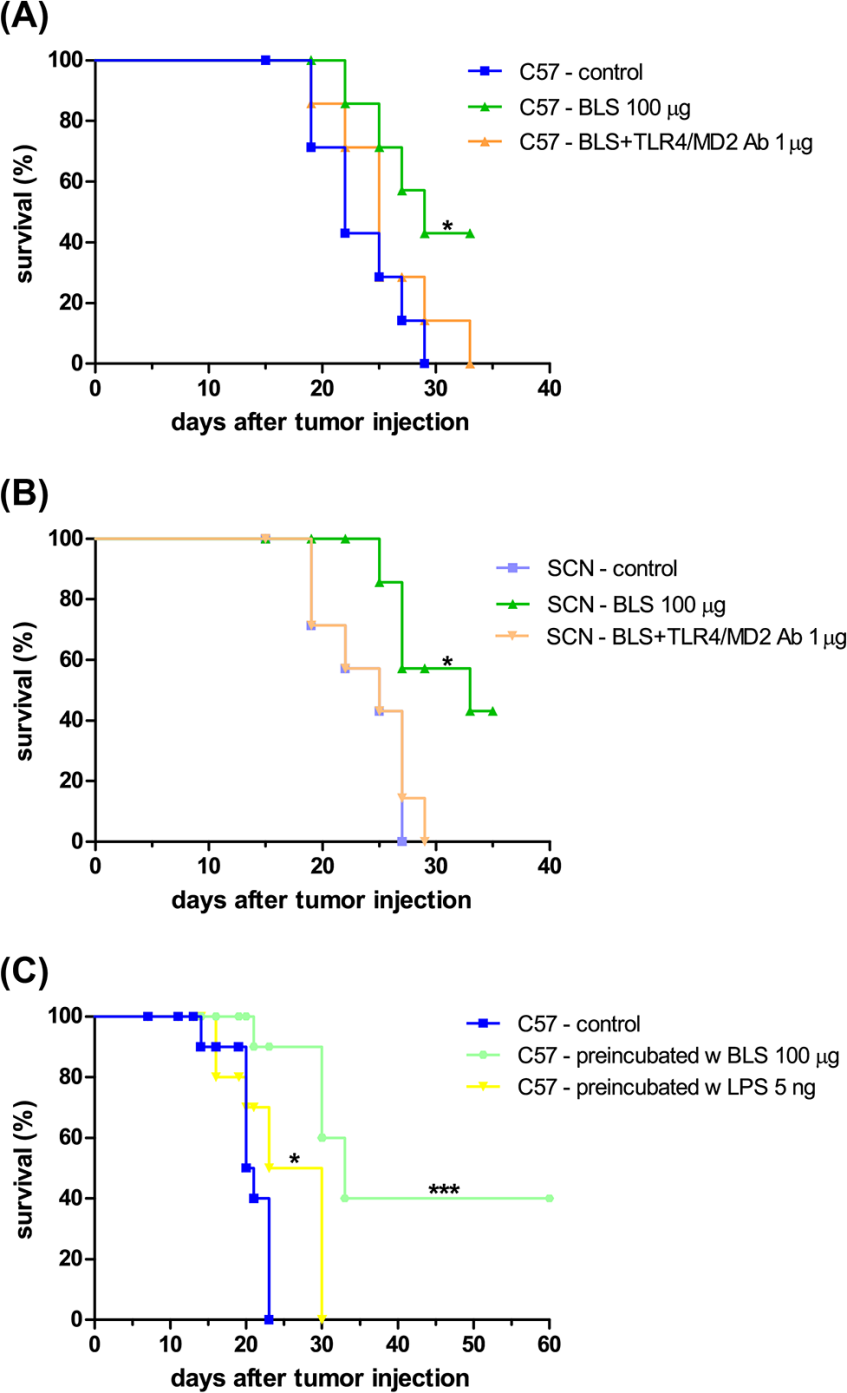
BLS signals B16 cells directly via TLR4. B16 cells were preincubated *in vitro* with 100 μg of BLS or 5 ng of LPS. After 48h cells were washed and 2.5x10^5^ cells were sc inoculated in the right flank of C57BL/6J mice (A). To block TLR4, cells were incubated with TLR4/MD2 monoclonal antibody; then they were stimulated with 100 μg of BLS (BLS+TLR4/MD2 Ab) and inoculated into C57BL/10ScNJ (B) and C57Bl/6J (C) mice. Figures show the survival rate; n = 7, * p<0.05, *** p< 0.001.

**Fig 6:**
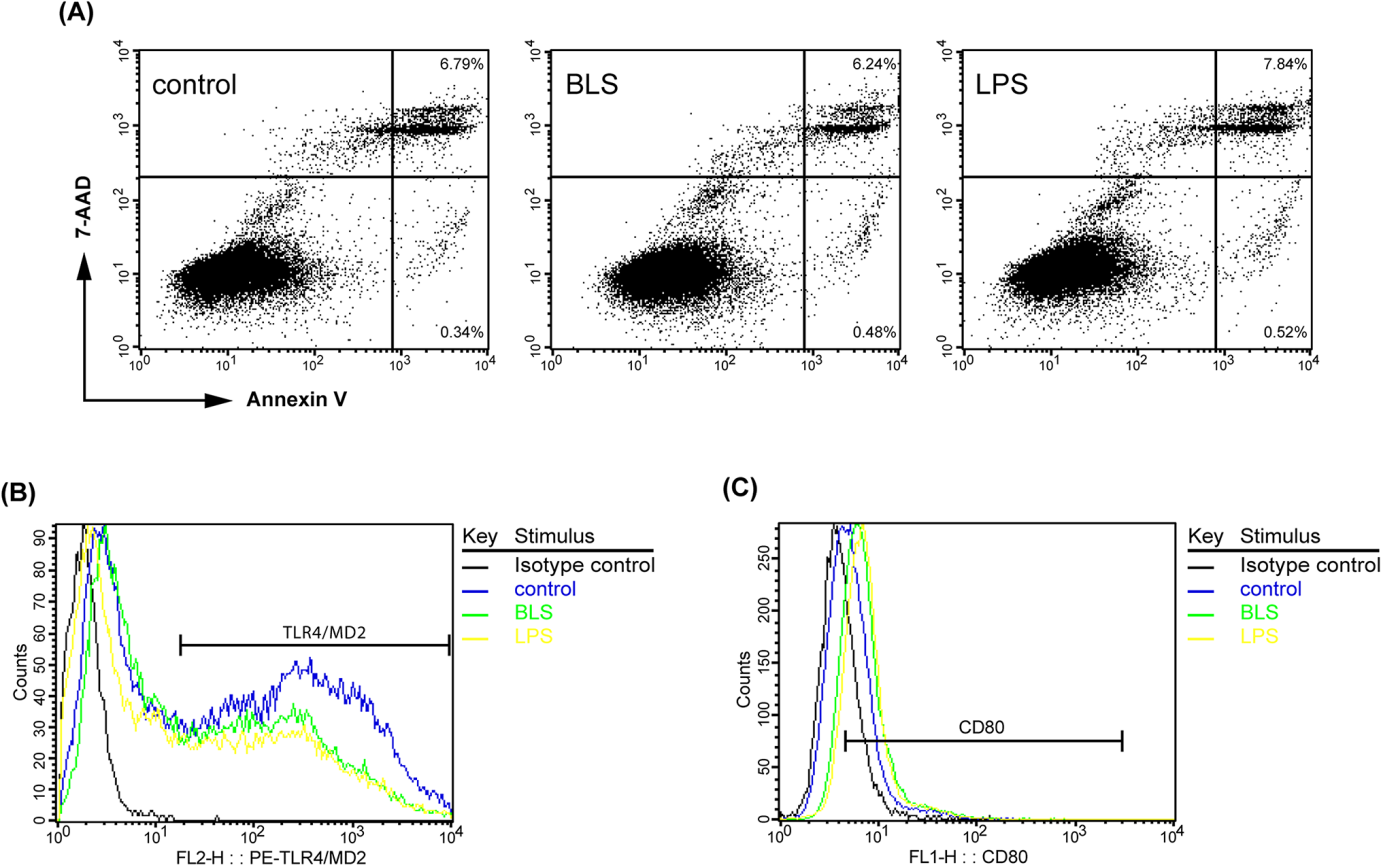
BLS direct effect on B16 cells. B16 cells were cultured in a 6-well plate (2.5x10^5^cells/well) in 2 mL standard cell culture medium with 100 μg of BLS or 5 ng of LPS for 48h. (A): Apoptosis was assessed by staining with Annexin V-PE/7-AAD and fluorescence-activated cell sorter analysis was performed. Representative dot plots of unstimulated (control), BLS- and LPS-stimulated B16 cells are shown. (B): Expression of surface TLR4/MD2 was analyzed by FACS in B16 melanoma. Results depict representative overlayed histograms of unstimulated (control) B16 cells, BLS- and LPS-stimulated cells. (C): Expression of CD80 in B16 melanoma was analyzed by FACS. Representative overlayed histograms are shown of unstimulated (control) B16 cells, BLS- and LPS-stimulated cells.

It was previously reported that *in vitro* stimulation of B16 cells with LPS reduces subsequent tumor growth in mice and that this effect is dependent on tumor TLR4 [67]. To study if BLS induces a similar effect, B16 cells were preincubated *in vitro* with BLS or LPS. After 48h, cells were washed and inoculated in C57BL/6J mice. Fig 5A shows the survival of mice injected with B16 melanoma preincubated with BLS, LPS or unstimulated B16 cells. Tumors induced by BLS-stimulated B16 cells had an inhibited growth compared to those induced by unstimulated B16 cells and also with LPS-stimulated B16 cells. Remarkably, in the BLS group 40% of mice did not develop tumors over a 120-day follow up. These results show that BLS has a direct effect in B16 cells that inhibits subsequent tumor growth. To evaluate if the TLR4 from recipient mice had a role in this effect, the same experiment was conducted in TLR4-deficient mice. As we expected, the extent of the inhibition of tumor growth induced by BLS prestimulation was similar in wild type and TLR4 deficient mice (Fig 5B). This result shows that the direct effect that BLS generates in B16 cells is independent on the presence of a functional TLR4 in mice. We then assessed the role of the TLR4 expressed in B16 cells, TLR4/MD2 monoclonal antibody was added to the cell culture prior to BLS stimulation. Results show that the inhibitory effect induced by BLS is completely abolished by blocking TLR4/MD2 in B16 cells (Fig 5B and 5C). These results clearly show that BLS triggers a mechanism in B16 cells via TLR4 that impacts in their subsequent growth *in vivo*. Analysis of the apoptosis levels in BLS-stimulated B16 cells, assessed by Annexin V/7-AAD staining and FACS analysis revealed that, as LPS, BLS did not induce programmed cell death (Fig 6A and Table 1).

**Table 1:** BLS direct effect on B16 cells.

Stimulus	TLR4/MD2^a^ ^,^ ^b^		CD80^a^ ^,^ ^b^	Annexin V & 7-AAD^a^ ^,^ ^b^			
	Positive	Hi	Positive	Annexin V (-) 7-AAD (+)	Annexin V (-) 7-AAD (-)	Annexin V (+) 7-AAD (+)	Annexin V (+) 7-AAD (-)
**control**	60.5 ± 4.1	37.0 ± 2.3	20.0 ± 3.0	02.4 ± 0.3	90.4 ± 1.4	06.8 ± 0.4	00.4 ± 0.0
**BLS**	48.0 ± 1.8	27.4 ± 1.2	47.7 ± 6.1	03.0 ± 0.5	90.3 ± 1.8	06.2 ± 0.4	00.5 ± 0.0
**LPS**	43.4 ± 6.1	24.7 ± 2.6	53.8 ± 6.0	03.0 ± 0.5	88.6 ± 1.1	07.8 ± 0.2	00.5 ± 0.0

^a^ (% ± SD)

^b^ n = 4

Finally, in a first attempt to address the mechanism involved in the direct effect induced by BLS in B16 cells, we measured the levels of surface molecules after BLS stimulation. The expression of TLR4 has been reported as either increased or decreased after LPS priming depending on cell types and experimental settings. It has been reported in several papers that B16 cells constitutively express TLR4 and that its level first increases upon LPS stimulation [67]. Therefore, we quantified the expression levels of TLR4/MD2 in B16 cells after 48h of stimulation with BLS or LPS. Fig 6B shows a representative histogram of TLR4 expression in non-stimulated and stimulated-with LPS or BLS- B16 cells. The expression of cell surface TLR4 is decreased in both stimulated groups to a similar extent. Quantification of CD80 expression levels revealed that BLS up-regulates this costimulatory molecule (Fig 6C), suggesting that B16 cells are activated upon BLS stimulation. The mean percentages of expression of TLR4 and CD80 are shown in Table 1. Further experiments are being conducted to reveal the mechanisms that could account for the protective effects.

Taken together, the results presented in this work show that BLS has a protective antitumoral effect in immunized mice and a direct effect in tumor cells. The effectiveness of the treatment with BLS prior to tumor cell inoculation depends on mice TLR4 signaling. In contrast, the therapeutic effect of BLS is independent of mice TLR4 and it is only achieved when mice are injected shortly after tumor cells are injected. Finally, we have shown that BLS impacts on B16 cells via TLR4 generating a subsequent diminished tumor growth. The therapeutic effect is probably due to the direct impact of BLS on tumor cells TLR4.

## Discussion

The use of TLR ligands in cancer therapy is an attractive approach that has been intensively studied in the past years in the context of cancer treatment or prevention. It has been demonstrated that TLR stimulation can lead to tumor regression either by direct induction of tumor cell apoptosis [68], reducing the proliferative capacity of tumor cells [67] or by activation of antitumor immune responses. Indeed, TLR stimulation can activate the innate immune response through the activation of NK cells, DC, or macrophages and the secretion of IFN-α, IFN-γ, and TNF-α [69–72] as well as the adaptive immune responses by favoring cross-presentation, Th1 polarization, and induction of cytotoxic T cells [73–75]. We have already described the characteristics of the immune response induced by BLS and have shown that it presents many of the desired attributes of an antitumor vaccine: (i) BLS activates DC, inducing the upregulation of costimulatory molecules and the secretion of cytokines like IL-12p70, and TNF-α; (ii) promotes the cross presentation of peptides and (iii) generates a rapid and strong *in vivo* specific cytotoxicity, being most of these effects regulated by TLR4. Altogether, the results presented in this work show that the response induced by BLS via TLR4 in vaccinated mice is able to protect them-at least in some extent- against tumor growth. This effect would be mainly due to the innate response that BLS generates, as it is not enhanced when a tumor antigen is coupled to its structure. The capacity of BLS to activate DC, inducing the secretion of proinflamatory cytokines and also activating B cells and T CD8+ cells via TLR4 may account for the observed effects in the preventive assays. We have also shown that BLS has the ability to induce the cross presentation of covalently linked OVA peptide [38] and we have recently observed that this antigen presentation continues for at least 24 days after immunization. We therefore hypothesized that immunization with the chimera BLS-OVA would generate a greater inhibition of OVA-expressing melanoma growth compared to immunization with BLS. Unexpectedly, no differences were observed in the protective effect of BLS and BLS-OVA against B16-OVA tumor, at least in the experimental conditions performed in this work. Treatment with BLS or BLS-OVA of mice inoculated with B16-OVA melanoma also induced similar results. Both proteins induced a delay in tumor growth and a significant increase in mice survival to a similar extent. Maybe boost immunizations would be required to achieve an effective specific response with a greater impact on tumor growth both in prophylactic and treatment vaccination protocols; shorter times between immunization and tumor inoculation could also give better results. In accordance with our results, Hodi et al. showed in a phase 3 study, that co-immunization with the melanoma antigen gp100 did not improve the effect of immunotherapy with the anti-CTLA-4 ipilimumab monoclonal antibody [76, 77]. Besides, they observed that the clinical response in the patients treated with ipilimumab had a correlation with the occurrence of immune-related adverse events. The mechanism underlying the clinical activity of CTLA-4 immunotherapy is currently under investigation, and recent experimental findings indicate that antibody-mediated depletion of regulatory T cells (Tregs) in the tumor microenvironment plays a key role in efficacious antitumor responses [78–80]. BLS and ipilimumab would constitute different immunoregulatory therapeutic strategies sharing the common feature that specific immunity does not enhance their effect.

To address whether BLS can stimulate tumor cells directly, B16 cells were incubated *in vitro* with BLS and after 48h were washed and inoculated into wild type and TLR4 deficient mice. Remarkably, BLS-stimulated cells had a delayed growth, with a complete inhibition of tumor growth in 40% of mice in both strains. We also showed that the effect induced by BLS is greater than the inhibition induced by LPS. Maccioni´s group reported that LPS signaling via TLR4 on tumor cells *in vitro* triggers the secretion of IFN-γ by tumor-infiltrating lymphocytes, and the secretion of IFN-β by tumor cells, modifying tumor outgrowth *in vivo* [67, 81]. Our previous results also show that immunization with one dose of 50 μg of BLS induces the expression of IFN-γ in DLN in a TLR4-dependent way [38]. It is likely that BLS induces these cytokines secretion and probably others, activating DC and CTL; the underlying mechanisms involved in the induced response remains to be studied. We can conclude that the direct effect induced by BLS in B16 melanoma is dependent on tumor TLR4 as this effect disappears when this receptor is blocked with a monoclonal antibody before stimulation. Another finding supporting the direct effect of BLS on tumor cells TLR4, is that the therapeutic effect correlates with the presence of TLR4 at the tumor´s surface. In this experimental model, TLR4 is expressed in most of the B16 cultured cells and its level is downregulated over time *in vivo*. This could explain the different outcomes of BLS treatment at day 2 or day 10 after tumor cells inoculation. Also, we have recently assessed whether melanin was present in B16 melanoma cells and tumors in mice. Melanin was absent in cultured B16-OVA and in tumors up to day 7 after inoculation. At day 10 after tumor inoculation, melanin was evidenced and its level increased over time between days 10 to 14. It has been reported that melanin can attenuate treatment efficiency of radio-,chemo-, photo-, and immunotherapy because: it has scavenging capabilities and inhibitory effects on lymphocytes [82], and that melanogenesis stimulates expression of HIF-1α, classical HIF-1-dependent target genes involved in angiogenesis and cellular metabolism, including glucose metabolism and stimulation of activity of key enzymes in the glycolytic pathway and several other stress related genes [83]. Melanogenesis could contribute to the lack of efficacy of BLS treatment at day 10; it would be interesting to further investigate whether the coadministration of BLS and an inhibitor of melanogenesis can induce an enhanced therapeutic outcome compared to BLS alone. As mentioned in the Introduction, Eiro et al [23] investigated the expression and clinical relevance of various TLRs in cutaneous malignant melanoma (CMM) from patients and found that high TLR4 expression in tumor (including tumor cells and stromal cells) was significantly associated with a shortened relapse-free survival. Therefore they state that TLR4 expression may be a prognostic factor of unfavorable evolution in CMM and postulate TLRs and their signaling pathways as potential therapeutic targets to control tumor progression in CMM. As BLS has a direct effect in B16 cells via TLR4, it is expected that BLS treatment would be successful only when TLR4 is expressed in most tumor cell population. It would be interesting to compare the effect of BLS in CMM with high or low TLR4 expression and evaluate whether BLS could benefit the therapy for patients with high-TLR4 CMM.

We have studied the subcellular localization of BLS by confocal microscopy in bone marrow-derived dendritic cells and our results show a colocalization between BLS and TLR4, first at the cell membrane and at later times with cytoplasmic TLR4 (unpublished). These results suggest that BLS binds to TLR4 at the plasma membrane and subsequently enters to the cytoplasm. We have also observed that the expression level of TLR4 first increases upon stimulation with BLS, then decreases and lately is reestablished to control levels, as assessed by flow cytometry. The shift in the surface expression level of TLR4/MD2 in dendritic cells has a correlation with the localization of BLS and TLR4/MD2 assessed by confocal microscopy. Our hypothesis is that BLS enters to the cytoplasm of dendritic cells with TLR4 and probably this receptor is recycled, returning to the cell membrane. A similar mechanism may be responsible for the decreased expression of TLR4 observed in BLS-stimulated melanoma cells *in vitro*. It would be interesting to study the evolution of TLR4 expression in the tumors of BLS-treated mice.

In summary, in this work we demonstrate that BLS elicits an antitumor immune response, resulting in slower tumor growth and longer survival of the tumor-bearing hosts. Our findings offer a new perspective on the antitumor effect of BLS that could lead to a therapeutic strategy utilizing a TLR4 ligand. As the expression of TLR4 has been reported on a large number of tumors, BLS signaling via TLR4 could make a notable contribution to the success of cancer treatment when coadministered with other cancer vaccines or treatments like radiation or chemotherapy.

## Supporting Information

S1 Fig BLS and BLS-OVA increase the level of CD86 in bone marrow dendritic cells. Expression of CD86 in CD11c+ BMDCs was analyzed by FACS after 18h of stimulation with BLS or BLS-OVA. Representative overlayed histograms are shown of unstimulated (control), BLS- and BLS-OVA-stimulated cells.
(TIF)
