# Supplementary figures and images for: Brucella spp. Lumazine Synthase Induces a TLR4-Mediated Protective Response against B16 Melanoma in Mice
Source: PLoS One. 2015 May 14;10(5):e0126827. doi: 10.1371/journal.pone.0126827 (PMC4431812; doi:10.1371/journal.pone.0126827)

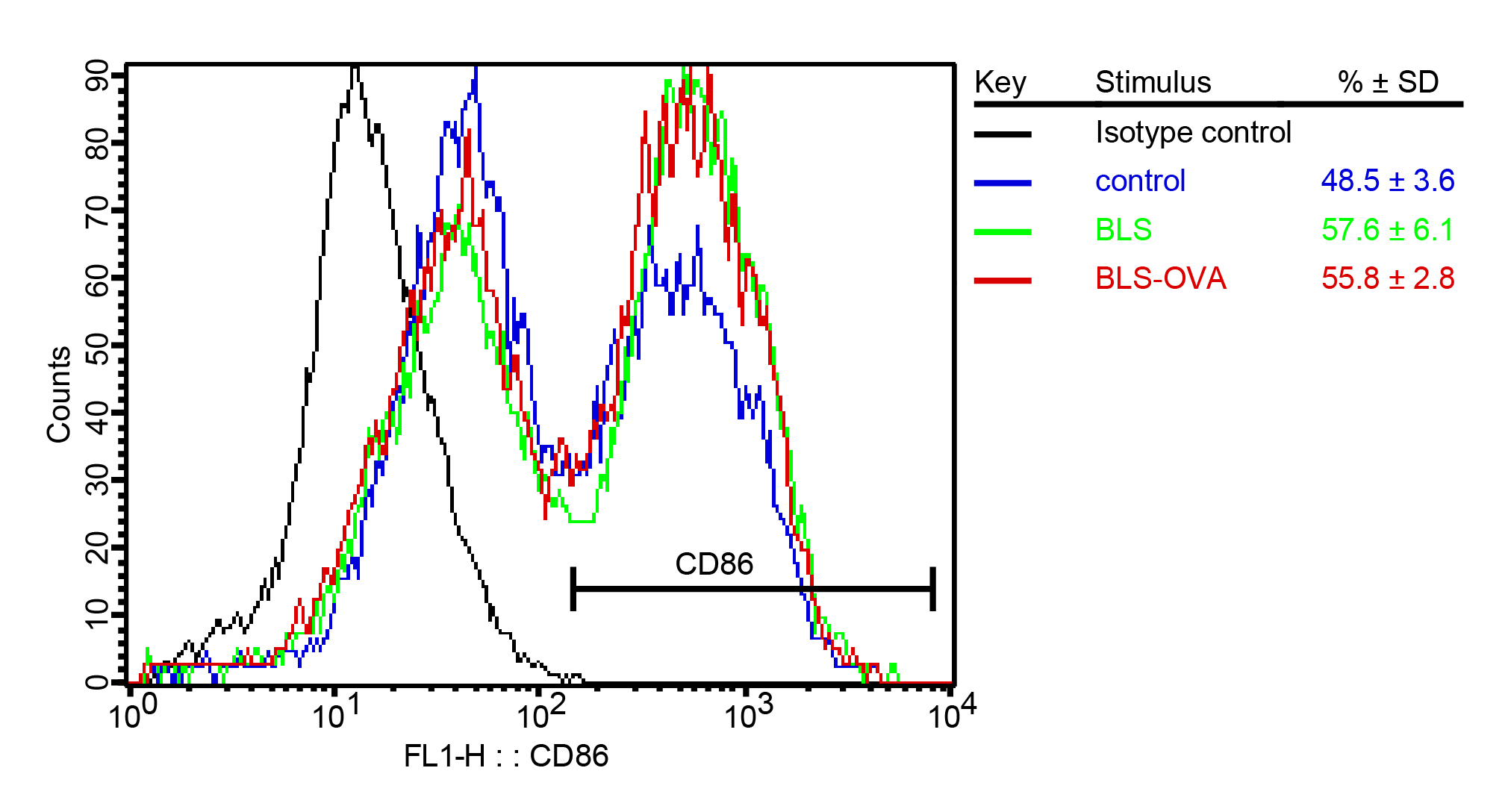

Supplement: S1 Fig — Expression of CD86 in CD11c+ BMDCs was analyzed by FACS after 18h of stimulation with BLS or BLS-OVA. Representative overlayed histograms are shown of unstimulated (control), BLS- and BLS-OVA-stimulated cells. (TIF) [file pone.0126827.s001.tif]
